# Evaluation of Patients' Education on Foot Self-Care Status in Diabetic Patients

**DOI:** 10.5812/ircmj.1138

**Published:** 2012-12-06

**Authors:** Parichehr Kafaie, Mohamad Taghi Noorbala, Sedigheh Soheilikhah, Maryam Rashidi

**Affiliations:** 1 Shahid Sadoughi University of Medical Sciences, Yazd, IR Iran

**Keywords:** Diabetic Foot, Education, Self Care

## Abstract

**Background:**

Skin problems caused by neuropathy and antipathy are common manifestations of diabetes. The most serious about such problem is the diabetic foot, which may lead to eventual ulceration and amputation, and will decrease a patient’s quality of life dramatically.

**Objectives:**

The aim of this study is to assess the level of foot self-care and foot conditions in diabetic patients, and to demonstrate the role of self-care education in diabetic foot care.

**Patients and Methods:**

A total of 80 diabetic patients were included in the study, all of whom had referred to "Yazd Diabetic Research Center." The levels of their foot self-care were recorded in pre-test questionnaires, and then all of the patients were visited and educated by a Dermatologist for their foot self-care on a monthly basis, after which their post-test results were recorded through a second administration of the same questionnaire. Eventually, data from the pre and post-test questionnaires were analyzed to identify the possible effects of education.

**Results:**

A total of 80 diabetic patients (34 males, 46 females) with a mean average age of 53.53 ± 10.19 and mean average duration of diabetes 12.42 ± 6.73 years were assessed. A significant increase in foot self-care through education was observed (baseline 27.06 ± 8.77, vs. post education 43.12 ± 8.77; P = 0.0001). After education, foot and nail lesions improved completely in 84% and 62.8%. Moreover, 77.8% of patients had suitable shoes and 79.6% had suitable socks.

**Conclusions:**

Our findings showed that foot self-care education could improve knowledge and performance of patients about various foot problems, and was significantly important in preventing ulcers.

## 1. Background

There are a high number of diabetic patients in the world, and this number is increasingly spreading. According to a study conducted by the World Health Organization (WHO) there will be around 300 million diabetic patients worldwide in 2025 (1). In Iran, the frequency of diabetes is reported to be 5 to 8 percent of the total population, with the highest occurrence rates in Yazd and Bushehr (2). Considering that this group of patients will likely suffer a variety of problems, it is essential that patients receive proper information about these probable problems. One common condition relates to skin and nail problems, which in association with neuropathy and vasculopathy leads to diabetic foot ulcer and patients' quality of life decreases dramatically with potential foot amputation. Diabetic patients, more than any other chronic diseases patients, require daily self-management.

## 2. Objective

The aim of this study is to assess the level of foot self-care and foot condition in diabetic patients, and to demonstrate the role of face to face education in diabetic foot care.

## 3. Materials and Methods

A total of 80 diabetic patients were enrolled in this quasi-experimental study, which had been referred to Yazd Diabetic Research Center. The level of their foot self-care was assessed by a self-induced questionnaire based on NICE guideline (3) before education, and then all the patients were visited and educated by a qualified dermatologist in regards with foot self-care on the same basis. The education method was face to face, which included teaching them the necessity of washing their feet, how to check their feet, drying between toes, using moisturizers, cutting their toenails properly, wearing slippers and inspecting the insides of their shoes. Education also included self-inspection of foot conditions, including skin texture, focal lesions (callus, cracks and pigmentation), inter-digital lesions (callus, maceration), nail problems (incurved, nail plate thickness, ingrown nails, discoloration, fungal infection). Foot deformity and footwear conditions were evaluated and recorded by a specialist. The patients were visited monthly for 3 consecutive months, and after that the questionnaire was reassessed. The pre and post-test questionnaires were analyzed to identify the effects of the education. For better analysis of the data, 3 columns of the table; daily, often and sometimes were assessed as acceptable and two other columns unacceptable. On the basis of 5- point liker scale (4) 1 to 5 scores has given for foot self-care behavior of patients. Total scoring varies from 12 to 60. Total score over 36 defined as adequate knowledge and others inadequate. The mean of knowledge before and after education was compared with "t-test". Complete Improvement means completely re-epithelization of the ulcer, and if the size of ulcer decreased at least 50% define as partially improvement.

### 3.1. Statistical Analysis

The all collected data were transferred directly into SPSS 17. Analysis of the data was performed, using Chi Square or other related tests for comparing the groups. P ≤ 0.05 was considered significant.

## 4. Results

Eighty subjects (34 males, 46 females) with mean average age of 53.53 ± 10.19 and mean average duration of diabetes 12.42 ± 6.73 years were assessed in the study. Table 1 shows the relevant variables before and after education. According to this study, the lack of adequate knowledge of diabetic foot care (total score under 36) was recorded to be 76.6%, not inspecting their feet 43.1%, improper clipping their toenails 67.3%, not washing their feet daily 60.4%, never using moisturizing creams 63.8%. High risk practices including walking barefoot were recorded at 86.2%, wearing shoes without socks 21.4%, not checking water temperature before bath 44.9%. In the baseline, 98.1% of subjects had focal lesion, 11.5% inter-digit lesion, 49.2% nail problems, and 23.8% foot deformity. After 3 months, foot and nail lesion improved completely in 83% and 62.8% of the cases, and improved partially in 16.4% and 22.8% of the subjects respectively. Moreover, 77.8% of patients had suitable shoes and 79.6% had suitable socks. All of foot self-care behavior in patients was improved after education and eventually 85% of patients had adequate knowledge. A significant improvement in foot self-care was observed in the patients after education according to our scorings (before 27.06 ± 8.77, vs. after 43.12 ± 7.37). The differences between observed values were tested (P = 0.0001).

**Table 1:** Foot Self Care Behavior Before and After Education

Foot Self-Care Behavior	Daily, %	Often, %	Sometimes, %	Rarely, %	Never, %
**Inspect Feet**					
Before	37.9	8.6	10.4	43.1	-
After	87.9	7.6	4.5	-	-
**Inspect Shoes Before Putting Them On**					
Before	13.8	12.1	12.1	10.3	51.7
After	53	18.2	15.2	1.5	12.1
**Wash Feet**					
Before	39.6	25.9	34.5	-	-
After	86.4	10.6	3	-	-
**Dry Feet Well After Washing**					
Before	-	17.2	20.7	13.8	48.3
After	-	68.2	21.2	3	7.6
**Use Emollients For Dry Skin**					
Before	13.8	19	3.4	63.8	-
After	71.2	19.7	3	6.1	-
**Cut Toenails Properly**					
Before	-	8.6	24.1	41.4	25.9
After	-	54.5	27.3	10.6	7.6
**Walk Bare Foot**					
Before	-	60.3	25.9	8.6	5.2
After	-	47	9.1	4.5	39.4
**Wear Shoes Without Socks**					
Before	-	10.7	10.7	78.6	-
After	-	6.1	3	3	87.9
**Check Between Toe**					
Before	24.1	22.4	25.9	27.6	-
After	86.3	6.2	4.5	3	-
**Dry Between The Toes**					
Before	-	17.3	22.4	15.5	44.8
After	-	72.7	7.6	6.1	13.6
**Check of Water Temperature Before Bath**					
Before	-	29.3	25.8	19	25.9
After	-	74.2	9.1	3	13.7
**Seek Professional Help For any Problem**					
Before	-	46.697	36.23	10.3	6.9
After	-	97	3	-	-

## 5. Discussion

Diabetes, late complications, and diabetic foot ulcers in particular, are of great importance due to the risk of amputations, reduced functioning, escalating financial burdens, and the dramatic reduction of the diabetic patient's quality of life due to possible occurrence of ulcers. As mentioned already, diabetes, more than any other chronic disease, requires daily self-management and this study demonstrates the role of face to face education in diabetic foot care improvement. Our findings show that face to face education is preventive and vitally important for diabetic patients. Other studies have similarly shown that educating patients, and the health services, are one of the most important contributors to the prevention of diabetic foot ulcer. In the study conducted by Litzelman et al., diabetic patients were educated for foot self-care, with a combination of telephone and postcard reminders helped these patients in improving their care conditions, the results were compared with usual care patients for 18 months (5). Lower serious foot lesions, higher average scores for self-reported care was reported, As well as in the Pieber study. Weekly sessions on Diabetes Mellitus education and foot care education compared with usual care showed significantly reduced callus formation and "poor nail care" compared with the base of the study (6). Ulcers which usually occur on the dorsal aspect of toes or on the bony eminences of the foot are often not due to trauma, and are more often caused by poorly fitting shoes. Thus, preventive care with footwear is very important (7, 8). A study carried out in the United Kingdom showed that the risk of foot ulcer is lower in Asian and African patients in compare with European diabetic patients (9). Other studies show that the risk of amputation in diabetic patients, who have suffered trauma to their feet, is 46 times more than non-diabetic patients, and it is important to consider that penetrating traumas in diabetic patients with bare foot is 2 times more than non–diabetic patients. These findings demonstrate the role of self–care education in diabetic foot care and footwear (10, 11). A study carried out at University of Pennsylvania (2009); found that it is efficacious to provide prognostic information for diabetic foot ulcers in a wound care setting using an administrative database (12).

In another study which was conducted in New York (2000), the internet was used as a tool for educating diabetic patients (13). In a study which was done in 2007, the treatment of diabetic foot ulcers in the home with video consultations was evaluated, and all of the patients expressed their satisfaction, and reported their confidence in this new way of treatment (14). Other studies have also shown the importance of education through telemedicine (15-17). A study at University of California (2002) showed greater patient participation has the potential to improve diabetic self-care because of the likely positive effect of patient satisfaction on adherence to treatment (18). These and many other similar studies show and confirm the important role of education in preventive self-care behaviors in diabetic patients.
